# CD-1 mice females recognize male reproductive success via volatile organic compounds in urine

**DOI:** 10.18699/VJGB-23-58

**Published:** 2023-09

**Authors:** A.S. Khotskina, E.L. Zavjalov, E.P. Shnayder, L.A. Gerlinskaya, S.O. Maslennikova, D.V. Petrovskii, M.N. Baldin, A.L. Makas, V.M. Gruznov, M.L. Troshkov, M.P. Moshkin

**Affiliations:** Institute of Cytology and Genetics of the Siberian Branch of the Russian Academy of Sciences, Novosibirsk, Russia; Institute of Cytology and Genetics of the Siberian Branch of the Russian Academy of Sciences, Novosibirsk, Russia; Institute of Cytology and Genetics of the Siberian Branch of the Russian Academy of Sciences, Novosibirsk, Russia; Institute of Cytology and Genetics of the Siberian Branch of the Russian Academy of Sciences, Novosibirsk, Russia; Institute of Cytology and Genetics of the Siberian Branch of the Russian Academy of Sciences, Novosibirsk, Russia; Institute of Cytology and Genetics of the Siberian Branch of the Russian Academy of Sciences, Novosibirsk, Russia; Trofimuk Institute of Petroleum Geology and Geophysics of the Siberian Branch of the Russian Academy of Sciences, Novosibirsk, Russia; Trofimuk Institute of Petroleum Geology and Geophysics of the Siberian Branch of the Russian Academy of Sciences, Novosibirsk, Russia; Trofimuk Institute of Petroleum Geology and Geophysics of the Siberian Branch of the Russian Academy of Sciences, Novosibirsk, Russia; Trofimuk Institute of Petroleum Geology and Geophysics of the Siberian Branch of the Russian Academy of Sciences, Novosibirsk, Russia; Institute of Cytology and Genetics of the Siberian Branch of the Russian Academy of Sciences, Novosibirsk, Russia

**Keywords:** chemical signals, dihydrofuran, GC/MS, 6-hydroxy-6-methyl-3-heptanone, mating preference, olfactory preference, reproductive success, хемосигналы, дигидрофуран, ГХ/МС, 6-гидрокси-6-метил-3-гептанон, половое предпочтение, ольфакторное предпочтение, репродуктивный успех

## Abstract

Sexual selection is considered as one of the leading factors of evolutionary development. In the conditions of incessant competition, specialized methods of attracting individuals of the opposite sex as well as criteria for assessing the quality of a sexual partner have been formed. In order for animals to rely on signaling from sexual partners, the signal must reflect the morpho-physiological status of animals. A high reproductive efficiency of male mice is a good advantage for mate selection and thus must be somehow demonstrated to potential mates. The aim of our study was to find out if male mice could demonstrate their reproductive efficiency through urine volatile organic compounds. The experiment implies cohabiting one male with two mature females for 6 days. The reproductive success of the male was assessed by the presence or absence of pregnant females. At the same time, naive females, who did not participate in reproduction, assessed the urine of the successful males as more attractive, which was expressed in shorter Latency time of sniffs in the Olfactory test. Using a rapid headspace GC/MS analysis, we have found volatile organic compounds (VOCs) in male urine that correlated with female behavior. It turned out that these substances are derivatives of mouse pheromone 6-hydroxy-6-methyl-3-heptanone. The amplitude of peaks corresponding to this pheromone correlated with the testosterone level in blood and the weight of preputial glands. The amplitude of peaks increased in males after mating with whom the females turned out to be pregnant. It is important to note that body weight, weight of testes, weight of seminal vesicles, weight of preputial glands, and plasma testosterone level alone are not reliable indicators of male reproductive success. Thus, the content of the pheromone 6-hydroxy-6-methyl-3-heptanone in the urine of males can serve as a good predictor of the quality of the male as a sexual partner for female CD-1 mice.

## Abstract (ru)

Половой отбор рассматривается в качестве одного из ведущих факторов эволюционного развития. В условиях постоянной конкуренции сформировались специализированные способы привлечения особей противоположного пола, а также критерии оценки качества полового партнера. Самцы, способные оставить наибольшее количество потомков, должны быть более привлекательными половыми партнерами, что требует от них каким-либо образом демонстрировать самкам свои преимущества. При этом ключевое условие для реализации такого отбора – это способность сигнала достоверно отражать информацию о физиологическом состоянии организма особи. У мышей одним из ведущих каналов передачи информации являются хемосигналы. Целью нашего исследования было выяснить, могут ли самцы мышей демонстрировать самкам свою репродуктивную эффективность через летучие органические соединения мочи. В ходе эксперимента самцу подсаживали двух половозрелых самок на шесть дней для размножения. Репродуктивный успех самца оценивали по наличию или отсутствию потомства. В то же время половозрелые самки, ранее не участвовавшие в размножении, оценивали мочу этих самцов. Оказалось, что более привлекательной была моча самцов, способных оставить потомство. Это выражалось в более коротком латентном времени обнюхивания самкой мочи самца в ольфакторном тесте. При помощи газовой хроматографии в моче самцов были обнаружены летучие органические соединения, которые коррелировали с поведением самок. Масс-спектрометрический анализ образцов мочи показал, что эти соединения являются производными феромона мышей 6-гидрокси-6-метил-3-гептанона. Амплитуда пиков, соответствующих данному феромону, коррелировала с уровнем тестостерона в крови и массой препуциальных желез и была выше у самцов с фертильными покрытиями. Важно отметить, что ключевые морфофизиологические показатели, такие как масса тела, масса семенников, препуциальных желез, семенных пузырьков, а также уровень тестостерона в плазме крови самцов, не отражали их способность оставить потомство. Таким образом, содержание феромона 6-гидрокси-6-метил-3-гептанона в моче самцов CD-1 может служить хорошим критерием их репродуктивной эффективности.

## Introduction

Animal olfactory cues contain a vast amount of information that plays an important role in their life and population processes. The odors play a special role in the relations between the sexes and ensure the process of mating (Brennan, Zufall, 2006; Arakawa et al., 2008). Most often, the rodents use urinary tags for information transfer (Hurst, Beynon, 2004). Urine could be considered as the body fluid with the highest capability to “yield” different volatile organic compounds (VOC) that could be used in chemocommunication (Novotny et al., 1999b). The complexity of mouse urine volatile profile has been described in a number of publications (Novotny et al., 2007; Schaefer et al., 2010). Through GC/MS analysis, more than two hundred VOCs were found on the chromatographic profiles of mouse urine, and for nearly half of them chemical structure was identified (Schwende et al., 1986; Jemiolo et al., 1987; Röck et al., 2007; Zhang et al., 2007; Schaefer et al., 2010; Liu et al., 2017). Several substances were described as unique mouse urine constituents, which are not present in urine of any other species – the mouse pheromones (Novotny et al., 2007). The biological activity of their majority has already been studied (Novotny et al., 1985, 1990, 1999b; Jemiolo et al., 1986). The role of other urinary volatile metabolites has been studied less than that of pheromones but they are also involved in the process of chemocommunication. Urinary metabolites form an odor background, which reflects individual features such as diet, stress level, genotype and others (Zhang et al., 2007; Schaefer et al., 2010).

Most known male pheromones, such as: 2-sec-butyl-4,5- dihydrothiazole and 3,4-dihydro-exo-brevicomine (Jemiolo et al., 1985), 1-hexadecanol and 1-hexadecanol acetate (Zhang et al., 2007), α- and β-farnesene (Jemiolo et al., 1991), MTMT (Lin et al., 2005), and darcin (Liu et al., 2017) are highly attractive for female mice. A lot of male pheromones, such as: 2-sec-butyl-4,5-dihydrothiazole, 3,4-dihydro-exo-brevicomine, α- and β-farnesene, 6-hydroxi-6-methyl-3-heptanone (Novotny et al., 1999a), and 2-isopropyl-4,5-dihydrothiazole have a stimulating effect on puberty in females (Osada et al., 2008). Wherein some of them: 2-sec-butyl-4,5-dihydrothiazole, 3,4-dihydro-exo-brevicomine (Jemiolo et al., 1986), α- and β-farnesene (Ma et al., 1999) can stimulate the estrus synchronization in female population (Whitten et al., 1968). Now it is known as Whitten effect. Beside this, all abovementioned pheromones induce the estrus cycle (Jemiolo et al., 1986; Ma et al., 1999).

Moreover, it was shown that the females of mice, rats, and voles could discriminate males. For examples, only by the scent of urine females can discriminate the genotype of males (Penn, Potts, 1998; Roberts, Gosling, 2003; Ilmonen et al., 2009; Manser et al., 2015), their maturity (Osada et al., 2003, 2008), hierarchy status (Drickamer, 1992; Veyrac et al., 2011), parasite load (Kavaliers, Colwell, 1995; Willis, Poulin, 2000), immunocompetence (Zala et al., 2004; Gerlinskaya et al., 2012), and infection status (Moshkin et al., 2001, 2002; Zala et al., 2015).

Now it is known that the free choice of a partner ensures the birth of the most viable offspring. In experiments on various species of animals it was shown that the survival rate from the moment of birth till reaching sexual maturity is significantly higher in individuals born when mating occurs in accordance with the free behavioral choice of a partner, compared to that when crossing contrary to choice (Drickamer et al., 2000; Gowaty et al., 2007; Nelson et al., 2013; Raveh et al., 2014). However, this result was obtained in experiments where females had direct contact with a partner by hearing, seeing and sniffing them. In these experiments wild-caught animals or their outbred offspring were used. To explain the positive effect of sexual choice, the hypotheses of “good genes” (Kokko, 2001), phenogenetic complementarity of the mother and father (Andersson, 2006), heterozygosity (Ilmonen et al., 2009), and Fisher’s “attractive sons” (Kokko, 2001) are used. The basis of all these theories is the choice of a partner based on his genotype. Theoretically, paternal effects may be associated with traits acquired during ontogeny and not dependent on genes, but this theory has not enough evidence at the moment. Therefore, for this study, we chose CD-1 mice, which have genetic diversity and are a frequently used model object of research.

In this study we attempt to determine whether the females will be able to recognize the successfully mated males only by urine tag, and, moreover, what kind of components detected in their urine by gas chromatography correlate with attractiveness of males for females in Olfactory test.

## Materials and methods

Mice and sample collection. We used 19 males and 86 (38 + 48) females of an outbred CD-1 mouse strain (2–3 months old) from the Centre for Genetic Resources of Laboratory Animals at the Institute of Cytology and Genetics, Siberian Branch of RAS (Novosibirsk, Russia) in this study. All animals had SPF-status.

The experiment was carried out in the spring-summer period. Mice were kept for 2 weeks in single-sex groups of four-five animals per standard cage (35 cm × 25 cm × 12 cm) with sawdust bedding at room temperature (20–22 °C) under a 14/10 h light/dark cycle (lights off at 18:00). Water and food pellets (Zoomir, St. Petersburg, Russia) were available ad libitum. Following the recommendation of Lombardi and Vandenbergh (Lombardi, Vandenbergh, 1977), we added soiled male bedding to female cages daily to support regular estrus cycles. Five days before the experiment males were placed into individual cages. On the day 1 of the experiment, 2 females were placed into each male cage at the time of lights off. Females were housed with males for 6 days, except for mated females (see below). Urine samples were collected on the 6th day. Urine was collected through gentle abdominal massage while the male was held over an Eppendorf microcentrifuge tube. The urine samples were divided into two aliquots, then immediately frozen and stored at –80 °C. Due to the fact that we did not spend more than 1 minute obtaining a urine sample from an individual mouse, only 16 out of 19 secondary aliquots were collected.

In procedures with animals, we used the principles specified in the European Convention for the Protection of Vertebrate Animals used for Experimental and Other Scientific Purposes. All animal protocols were approved by the Institutional Bioethics Committee of the Institute of Cytology and Genetics (No. 81).

Male reproductive success. Two females housed with males during 6 days were examined daily 2–3 hours after the lights were turned on for the presence of vaginal plugs. Females with vaginal plugs were removed and housed individually. After 6 days males were removed and sacrificed to assess the testosterone level in blood, body weight, weight of testes, seminal vesicles and preputial glands. Seminal vesicles were removed and weighed together with coagulation glands. A male was considered reproductively successful if at least one female kept with him turned out to be pregnant. We got 7 successful and 9 unsuccessful males. The time of cohabiting a male with females was chosen in accordance with (Gerlinskaya et al., 2012). Since in 96 % of females that have access to the smell of a male, the length of the estrous cycle does not exceed 5.5 days (Jemiolo et al., 1986), within 6 days, a female introduced at any stage of the cycle will be in oestrus at least once, which is necessary for fertile mating.

Olfactory test. Separate females, who did not participate in reproduction, were tested. In behavioral testing, a preliminary acquaintance with the smell of contaminated bedding can have a decisive effect on the ability of females to recognize and increase interest in the volatile components of male urine (Moncho-Bogani et al., 2002). Therefore, bedding contaminated by males was added to the cages of females on daily basis. The day before the experiment, the females were placed individually and the test was performed in a home cage. Urine was thawed for 20 minutes at room temperature. 20 μl of urine were applied to filter paper and placed in a vial (a single-use 5 mm truncated tip of an automatic pipette). That is why the females had access only to the volatile compounds of urine. The tip was fixed to the mesh lid of the cage in the corner. Females observed one accidentally selected urine sample (Dougherty, Shuker, 2015; Dougherty, 2020). 16 samples were examined. During the 10-minute test, the number of approaches to sniff (number of sniffs), the time spent sniffing the sample in seconds (total time of sniffs) and the time of the first approach to the stimulus in seconds (latency time of sniffs) were taken into account. After the test, a swab was taken from the females to determine the stage of the estrous cycle. A sample from each male was tested on 3 females, the data on their testing were averaged for further calculations (or Repeated measure ANOVA was used if specified). The stage of the cycle had a significant effect on the behavior of females: at the proestrus stage, the females approached the urine samples significantly earlier than the females at the diestrus stage ( p = 0.039, LSD test). There were no significant differences in other stages and no effect on other types of behavior. To exclude the influence of the cycle stage on further statistical analysis, residual variances were used.

Preparation of urine samples and concentration procedure. Mouse urine was thawed and 20 μl of urine from each male was then transferred to 7 ml glass vials with caps containing gastight PTFE/Silicone septum (Supelco). The vials with urine were then heated for 15 min at 40 °C for equilibration, and also for denaturation of urinary proteins that bind some volatiles. Immediately after the heating sample headspace was concentrated on 6 mg of Tenax (Chrompack, Netherlands) using special sorbent traps designed for EKHO- A-PID gas chromatograph (IPGG SB RAS, Novosibirsk, Russia). For concentration we used filtered air flow at flow-rate of 40 ml/ min. In total we pumped 80 ml urine headspace and air mix through the sorbent layer.

The samples were thawed and prepared consistently one after another with 20 min intervals, so preparation of samples was done consecutively so each sample was run on the Gas Chromatograph (GC) within 20 min (including 15 min of heating) after defrosting. Filtered air samples were routinely run as a control.

GC analysis of urine VOCs and data preparation. An EKHO-A-PID gas chromatograph with original software (Sorbat, IPGG SB RAS, Novosibirsk, Russia) was used in our study. 19 samples of mouse urine were run in the GC using the non-polar GC column (polidimethylsiloxane polycapillary, n = 920 capillaries) SE-30 22 cm × 0.6 mm with 40 μm coating (IPGG SB RAS). The temperature of the column was constant at 50 °C during the whole separation. The temperature of the injection port was set at 180 °C. Filtered ambient air with a constant flow-rate of 20 ml/min was used as a carrier gas throughout the analysis. The duration of each analysis was set at 300 s. For further statistical calculations, we used the amplitudes of the peaks.

MS analysis. Urine samples were treated on a custom-made GC/MS system (IPGG SB RAS, Novosibirsk, Russia), specialized for fast VOC analysis in air with a non-polar column HP-5 similar to SE-30 (Makas, Troshkov, 2004). Heated urine headspace was concentrated on Tenax as described above. A non-polar column HP-5, 15 m × 0.32 mm with 1 μm film (Agilent technologies, USA), was used for separation. The temperature of the column was constant at 45 °C for 5 minutes, then it was programmed with the rate of 10 °C/min to 150 °C. The temperature of the injection port was set at 280 °C. Helium was used as the gas-carrier with a flow-rate of 2 ml/min.

The operating parameters for the mass spectrometer were set as follows: scan rate 0.5 s from 45 to 250 m/z; ion source temperature set to 180 °C, with electron impact ionization energy at 70 eV. Identification of compounds was performed using NIST/EPA/NIH libraries (ver. 2.0.2008) and information from literature (Schwende et al., 1986; Novotny et al., 2007).

The GC/MS data analysis was performed by the AMDIS program (NIST, USA).

Additionally retention times of several standard compounds were analyzed on both columns to use them as reference points in subsequent procedures. The standard compounds were purchased from Acros Organics (Belgium) and Sigma- Aldrich (USA). For each peak in question we found reference compounds among pure chemicals to make the retention time of the reference compound as close as possible to the retention time of the target peak. Thus, we were able to surround the target peaks on the chromatogram with one or two reference points. For this purpose benzene, toluene, m-xylene, 2,5-dimethylpyrazine and nonane were chosen. Thus, we were able to confine the intervals where the target peaks could be found. Next, we compared the area and the amplitude of the peaks detected in the localized intervals with the same parameters of the target peaks obtained on EKHO-A-PID and found three peaks (RT 4.2, RT 7.9 and RT 87.7) caught on GC/ MS that satisfied all requirements. We identified the peaks as three dihydrofuran (DHF) derivatives – the dehydratation products of lactol – using data reported in the literature. The characteristic losses of m/z 126, m/z 111, m/z 97, m/z 83, m/z 69, and m/z 57 were identical to those obtained in the earlier studies with synthetic analogues of cyclic enol ethers (Novotny et al., 2007). Moreover, the ratio of target peaks in our study (1 : 0.32 : 0.12) was nearly the same as the ratio of cyclic enol ethers (1 : 0.30 : 0.10) in mouse urine calculated in one of the studies mention above (Harvey et al., 1989). This provides additional confidence that our target compounds were dihydrofuran derivatives: 5,5-dimethyl-2-ethyl-4,5- DHF, E-5,5-dimethyl-2-ethylenetetrahydrofuran, and Z-5,5-dimethyl- 2-ethylenetetrahydrofuran.

Statistical analysis. To analyze behavior, we used Repeated measure ANOVA, since each male sample was tested by 3 females. To compare the groups by the content of components, we used One way ANOVA. We used Spearman correlation coefficient to count correlation ratios in this study. No data were removed from calculations. All experimental data were obtained blindly, the belonging of the animals to the groups was indicated only at the stage of data analysis. The level of significance used was p <0.05.

## Results

**Fig. 1.**
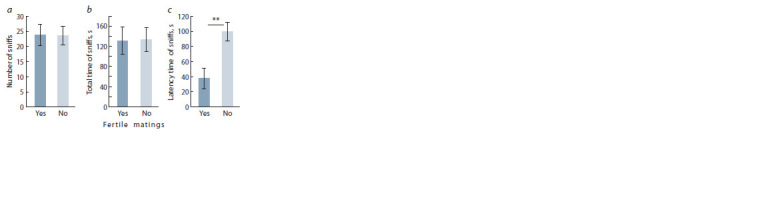
Behavioral reaction of naive females on smell of urine of male mice with various number of fertile matings: a, number of sniffs; b, total time of sniffs; c, latency time of sniffs. N(No) = 9 ; N(Yes) = 7 ; ** p < 0.01, Repeated measure ANOVA.

In Olfactory test, females had a shorter latency time of sniffs when studying urine samples from males who had already successfully procreated offsprings after 6 days of being kept with other females F1,14 = 0.00, p = 0.963 (Fig. 1, c). The presence of fertile matings in a male did not have a significant effect on the number of sniffs (F1,14 = 0.00, p = 0.963) and total time of sniffs (F1,14 = 0.00, p = 0.945).

Chromatographic study of volatile components of male urine samples revealed 12 peaks (Table 1). To understand whether the behavioral response of females is really related to differences in the content of the detected components, we performed a correlation analysis of the behavior characteristics and the obtained peaks of urine. A significant negative correlation was found between the latency time of sniffs and the amplitude of peaks RT 7.9 and RT 87.7 (see Table 1). No significant relationship was found between the amplitude of the peaks and the number of sniffs, as well as the total time of sniffs.

**Table 1.**
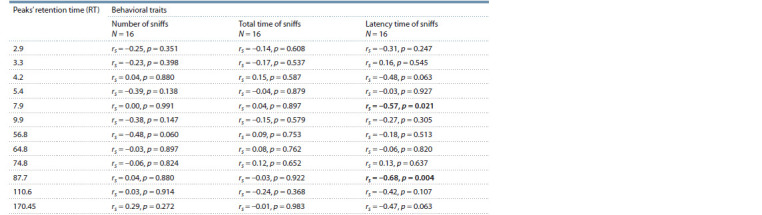
The relationship between the response of females in Olfactory test and the amplitude of chromatographic peaks in the urine of males Significant differences are given in bold.

Mass spectrometric analysis of male urine samples showed that the RT 7.9 peak corresponds to the known compound E-5,5-dimethyl-2-ethylenetetrahydrofuran and the RT 87.7 peak corresponds to Z-5,5-dimethyl-2-ethylenetetrahydrofuran. At the same time, one more dihydrofuran derivative was identified: 5,5-dimethyl-2-ethyl-4,5-DHF, RT 4.2. This compound did not show a significant correlation value with the behavioral characteristics of females (see Table 1). All three compounds are derivatives of the known male mouse pheromone 6-hydroxy-6-methyl-3-heptanone (HMH) (Harvey et al., 1989).

**Fig. 2.**
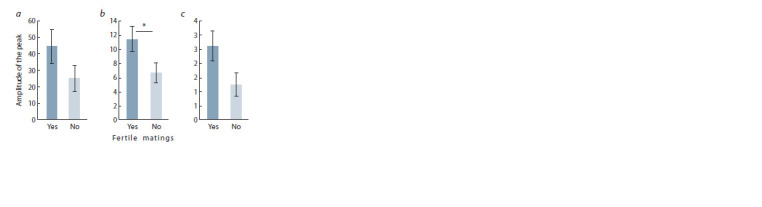
Amplitude differences in peaks RT 4.2 (a), RT 7.9 (b) and RT 87.7 (c) found in the urine of male mice that had or didn’t have fertile mating N(0) = 12 ; N(1) = 7 ; * p < 0.05, One way ANOVA

**Fig. 3.**
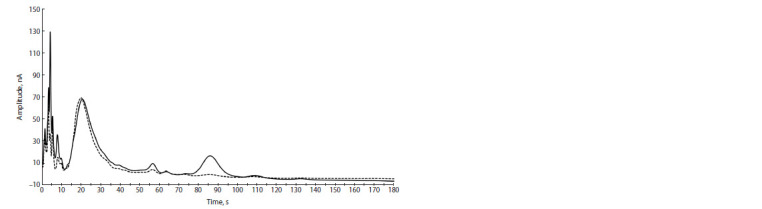
Sample chromatograms from a male with fertile mating (solid line) and without fertile mating (dotted line).

We evaluated the relationship between the amplitude of the chromatographic peaks of dihydrofurans and the reproductive success of males. The amplitude of the RT 7.9 peak was found to be significantly higher in the urine of males who had fertilized at least one female, compared to the urine of males who had fertilized no females (Fig. 2, Fig. 3). The RT 4.2 and RT 87.7 peaks showed the same trend, but the p-value was below the threshold of statistical significance (F1,17 = 2.19, p = 0.157 and F1,17 = 4.20, p = 0.056, respectively).

Analysis of the relationship between the amplitude of the three peaks under study and the level of testosterone in the blood plasma showed significant positive correlation of the amplitude of the RT 4.2, RT 7.9 and RT 87.7 peaks. A similar correlation was observed for the relationship between preputial gland weight and the RT 4.2 and RT 7.9 peaks. Correlation analysis of the studied peaks with body weight, weight of seminal vesicles and testes showed no significant values (Table 2).

**Table 2.**
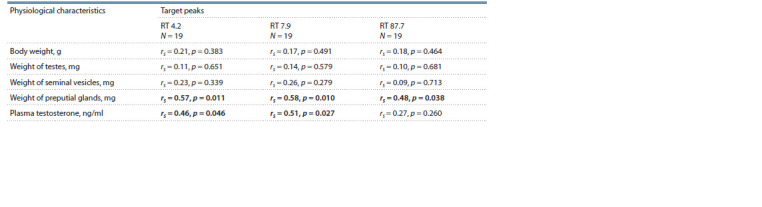
Correlation between physiological characteristics, testosterone level and amplitudes of target peaks (Spearman correlation coefficients) Significant differences are given in bold.

It is important to note that body weight, weight of testes, seminal vesicles, preputial glands, and plasma testosterone level alone are not reliable indicators of male reproductive success (Table 3).

**Table 3.**
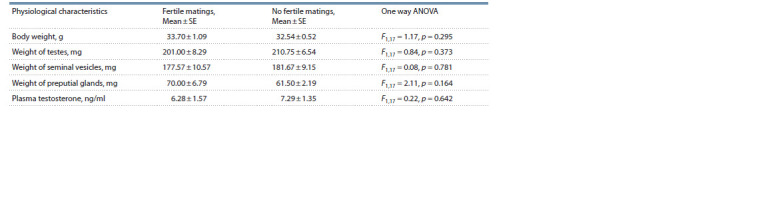
Mean values of physiological characteristics depending on the reproductive success of males

## Discussion

Two decades ago, it was shown for the first time that females can identify males, mating with which leads to greater reproductive success (Drickamer et al., 2000). This result has been repeated many times in different animal species (Drickamer et al., 2003; Gowaty et al., 2007; Nelson et al., 2013; Raveh et al., 2014). Nevertheless, direct contact with a partner and free access to all signals from a potential sexual partner in these experiments did not allow getting closer to the understanding of the selection mechanisms. In the present study, we used only volatile compounds of the male urine to be tested by females, and found that based on the smell of urine only, females could identify reproductively effective males. It turned out that the experimental females in Olfactory test approached the urine of successful males earlier (see Fig. 1, c) despite the fact that they performed an equal number of sniffs and had the same total time of sniffs (see Fig. 1, a, b).

The search for markers of male reproductive efficiency using gas chromatographic analysis of male urine samples showed that the behavior of females correlated with the amplitude of the dihydrofuran peaks (see Table 1)

Previously, DHFs have already been detected in significant amounts in the chromatograms of male mouse urine (Schwende et al., 1986; Jemiolo et al., 1987; Harvey et al., 1989; Novotny et al., 1999b). While studying the origin of these cyclic enol ethers in mouse urine S. Harvey et al. (Harvey et al., 1989) showed that DHFs originate from the tautomeric mixture of 6-hydroxy-6-methyl-3-heptanone and lactol via dehydratation in the inlet port of gas chromatograph under high temperature. It turned out that DHFs are not presented in the mouse urine by themselves, but their peaks in the chromatogram reflect the content of their precursor 6-hydroxy-6-methyl-3-heptanone (Harvey et al., 1989). Mutual precursors of target compounds explain very high coefficients of intercorrelation between these components, exceeding 0.90 ( p < 0.001). Thus, the behavior of females correlated with compounds that were previously shown to reflect the content of 6-hydroxy-6-methyl-3-heptanone in urine of males.

When studying the effects of HMH, it turned out that 6-hydroxy- 6-methyl-3-heptanone interacts with vomeronasal receptors (Del Punta et al., 2002), and therefore can trigger behavioral and physiological responses in females. HMH is known as a male mouse pheromone that accelerates puberty in female mice (Novotny et al., 1999a). Here we demonstrated that quantity of HMH in urine of a male reflects its ability to make fertile matings (see Fig. 2). Males with a lower level of this pheromone did not mate any of the two females for 6 days of joint maintenance. Perhaps exactly this indirectly explains the effect of a decrease in fertile matings in aged male mice, as shown earlier (Parkening et al., 1988), since HMH decreases in aged males (Osada et al., 2008; Varshavi et al., 2018), and in the work of Schaefer with colleagues HMH has been associated with the maturation state (Schaefer et al., 2010). In our work, when analyzing correlations with androgen-dependent characteristics of males, it turned out that amplitudes of the peaks correlated positively with plasma testosterone and weight of the preputial glands (see Table 2). However, reproductive success was not directly related to physiological characteristics of males (see Table 3). Taken together, these data show that 6-hydroxy-6-methyl-3-heptanone reflects reproductive quality of male mice.

On the other hand, it has previously been shown that HMH does not affect the attractiveness of male urine samples to females (Osada et al., 2008). These conclusions about male attractiveness were based only on the total time of sniffs, which is consistent with our results. We also demonstrated no relationship between the level of this pheromone in urine and the total time of sniffs (see Table 1). In addition to this, when assessing behavioral response of females to the stimulus, we found that the HMH content correlated with the latency time of sniffs (see Table 1), which indicates the importance of assessing other parameters of the females’ behavior, and not just the total time of sniffs.

## Conclusion

Identification of markers of reproductively successful males is an important task. Its solution does not have only fundamental importance, but will also allow targeted selection of more successful males when breeding animals. Urine, which is an accessible and unlimited resource, is of greatest interest in this regard. The observed relationships between reproductive success and the HMH content in urine require further research to understand the dynamics of this compound excretion at different stages of male ontogenesis, with different reproductive experience, in different conditions of social and microbiological environment.

## Conflict of interest

The authors declare no conflict of interest.
